# GenAI Use and GenAI-Assisted Learning Procrastination in University Students: The Roles of Planned Behavior Constructs and Learning GenAI Anxiety

**DOI:** 10.3390/bs16071256

**Published:** 2026-07-22

**Authors:** Zilin Li, Jiali Huang, Zhaodi Cui

**Affiliations:** Center for Teacher Education Research of Beijing Normal University, Key Research Institute of Humanities and Social Sciences for Universities, Ministry of Education (Institute of Teacher Education, Faculty of Education, Beijing Normal University), Beijing 100875, China; 202431010070@mail.bnu.edu.cn (Z.L.);

**Keywords:** GenAI, GenAI-assisted learning procrastination, Theory of Planned Behavior, learning GenAI anxiety

## Abstract

The rapid proliferation of generative artificial intelligence (GenAI) has reshaped university students’ learning practices, yet the association between GenAI-assisted learning and procrastination remains insufficiently understood. Drawing on the Theory of Planned Behavior as an established framework, this study offered a contextual extension of TPB and procrastination research into the GenAI domain by examining the association between students’ use of GenAI for learning task completion and GenAI-assisted learning procrastination among 1243 Chinese university students. Path analysis showed that students’ use of GenAI was negatively associated with GenAI-assisted learning procrastination (β = −0.195, *p* < 0.001). Behavioral attitude (β = −0.164, *p* < 0.001), subjective norm (β = −0.171, *p* < 0.001), perceived behavioral control (β = −0.331, *p* < 0.001), and behavioral intention (β = −0.137, *p* < 0.001) were each negatively associated with GenAI-assisted learning procrastination. The indirect associations linking GenAI use to GenAI-assisted learning procrastination through these Theory of Planned Behavior constructs were statistically significant, with the total indirect association accounting for approximately 30.9% of the total association (standardized indirect association = −0.086, *p* < 0.01). Learning GenAI anxiety moderated the association between behavioral intention and GenAI-assisted learning procrastination, with the negative association being stronger among students with higher anxiety (b = −0.268, SE = 0.040, *p* < 0.001) than among those with lower anxiety (b = 0.006, SE = 0.052, *p* = 0.915). The model explained 71.6% of the variance in procrastination in GenAI-assisted learning. Given the cross-sectional design and the sample’s specific characteristics, these findings should be interpreted as conditional associations rather than causal evidence, and their generalizability requires further investigation. Nevertheless, the results highlighted the roles of beliefs, intentions, and emotional experiences in understanding procrastination in GenAI-assisted learning.

## 1. Introduction

Learning procrastination, a common maladaptive behavior among university students, has long attracted research attention. Procrastination generally refers to the deliberate delay in initiating or completing tasks that one ought or intends to complete (Steel, 2007). While procrastination is a recognized issue across the general adult population, with approximately 20% identified as chronic procrastinators (Amarnath et al., 2023), research consistently suggests that its prevalence is significantly elevated among university students (Howell et al., 2006; Klingsieck et al., 2013). Procrastination is associated with diminished academic achievement (Araya-Castillo et al., 2023; Sæle et al., 2017; Sparfeldt & Schwabe, 2024) and with psychological distress, including heightened anxiety and depressive symptoms (Alaya et al., 2021; Jochmann et al., 2024; Steel & Klingsieck, 2016).

At the same time, the rapid development of generative artificial intelligence (GenAI) has substantially reshaped students’ learning environments. Since the introduction and widespread adoption of tools such as ChatGPT, GenAI has attracted increasing attention in educational research and practice (Rahman & Watanobe, 2023; Rejeb et al., 2024; Shahzad et al., 2024). GenAI tools can generate text, images, and videos in response to conversational prompts, thereby providing students with rapid and flexible support for learning-related tasks (Țală et al., 2024). The Artificial Intelligence Index Report 2025 from Stanford University’s Human-Centered Artificial Intelligence notes that advances in efficiency, affordability, and accessibility have lowered the barriers to using advanced AI and accelerated its adoption in professional, educational, and everyday contexts (Maslej et al., 2025). In higher education, students may use GenAI to retrieve information, ask questions, access learning resources, generate ideas, and obtain personalized support beyond temporal and spatial constraints (Bhullar et al., 2024; Rudolph et al., 2023).

GenAI offers unprecedented opportunities for personalized and efficient learning. However, its unreflective use poses risks to academic integrity and cognitive engagement. These competing considerations raise an important question for university learning: how is students’ use of GenAI (SUG) associated with procrastination in completing learning tasks? Two opposing mechanisms may underlie this association. On one account, GenAI may reduce the perceived difficulty of task initiation by providing immediate assistance, feedback, and idea generation (Stojanov, 2023; Strzelecki, 2023). On another account, GenAI use may also create new risks—such as overreliance, superficial engagement, academic misconduct, or avoidance of cognitively demanding work—that could exacerbate procrastination (Abbas et al., 2024; Cui et al., 2025). These negative aspects suggest that the educational value of GenAI use depends critically on how, when, and why students employ these tools—highlighting the need to examine the psychological processes that shape students’ engagement with GenAI in learning tasks. Therefore, the association between GenAI use and procrastination should not be understood as uniformly beneficial or detrimental. Rather, it requires a more nuanced examination of the psychological beliefs, intentions, and emotional experiences that are associated with students’ engagement with GenAI in learning tasks.

Previous research has primarily focused on students’ perceptions, acceptance, and perceived usefulness of GenAI (Cabrera & Neville, 2025; Katsantonis & Katsantonis, 2024), with limited attention to how students’ use of GenAI for specific learning tasks is associated with procrastination in GenAI-assisted learning task completion. From the perspective of intention–behavior consistency, procrastination can be understood as a failure to translate intention into timely action. Among university students, this may manifest as delays in daily assignments, final-term tasks, and examination preparation, despite a genuine intention to complete them. This feature of procrastination aligns closely with the Theory of Planned Behavior (TPB), which emphasizes the role of behavioral attitude (BA), subjective norm (SN), perceived behavioral control (PBC), and behavioral intention (BI) in explaining behavior-related outcomes (Ajzen, 1991). Within the TPB framework, the focal behavior must be clearly specified so that behavioral attitude, subjective norm, perceived behavioral control, behavioral intention, and behavior outcomes refer to the same target behavior. In this study, the focal behavior is defined as GenAI-assisted learning task completion, namely, students’ use of GenAI to support daily assignments, final-term assignments, and examination preparation. Accordingly, all TPB constructs are conceptualized in relation to this specific behavior. In this study, GenAI-assisted learning procrastination (GALP) is specifically defined as the unnecessary delay in initiating or completing the use of GenAI to support learning tasks, despite holding a genuine intention to use the technology and being aware of the potential costs of such delay. This specification distinguishes GALP from the general non-use or conscious avoidance of GenAI by focusing on the intention–behavior gap in the context of GenAI-assisted learning. This specification ensures consistency among the target behavior, its related beliefs and intentions, and the procrastination outcome. This study does not aim to propose a new theoretical model. Rather, it offers a contextual extension of TPB and procrastination research into the GenAI domain, providing initial empirical evidence on these relationships within a specific higher education context.

However, there is a substantial gap between behavioral intention and actual behavior, indicating that intention alone does not guarantee subsequent action (Sheeran & Webb, 2016). In the context of GenAI in education, AI-related anxiety has emerged as a growing concern. University students’ learning GenAI anxiety (LGA) is negatively associated with their actual learning behaviors (Y. Y. Wang & Wang, 2022). Therefore, learning GenAI anxiety may be a relevant boundary condition for the association between students’ behavioral intention and procrastination in completing GenAI-assisted learning tasks. This study thus examines not only the indirect associations of TPB constructs but also the moderating role of learning GenAI anxiety in the association between behavioral intention and GALP.

The research questions are as follows:

**RQ1:**  *How is students’ use of GenAI associated with procrastination in GenAI-assisted learning task completion among Chinese university students?*

**RQ2:**  *Do behavioral attitude, subjective norm, perceived behavioral control, and behavioral intention show indirect associations with students’ GenAI use and procrastination in GenAI-assisted learning task completion?*

**RQ3:**  *Does learning GenAI anxiety moderate the association between behavioral intention and procrastination in GenAI-assisted learning task completion?*

Given the cross-sectional nature of the data, all associations examined in this study are interpreted as conditional associations rather than causal.

## 2. Literature Review and Hypotheses

### 2.1. Prior Studies and Research Gaps

The literature on GenAI in education has grown rapidly, with substantial empirical advances in several areas. However, alongside these advances, researchers have increasingly raised concerns about the negative aspects of GenAI use. A growing body of evidence suggests that students may develop overreliance on AI-generated content, which may be associated with diminished critical thinking, reduced cognitive effort, and superficial engagement with learning materials (Abbas et al., 2024; Cui et al., 2025). Concerns have also been raised about academic integrity, as the ease of generating coherent text with GenAI may blur the boundaries between original and AI-generated work (Rahman & Watanobe, 2023; Sullivan et al., 2023). Furthermore, researchers have cautioned that uncritical use of GenAI may inadvertently encourage avoidance of cognitively demanding tasks, as students may turn to AI-generated solutions rather than engage in independent problem-solving (Chan & Hu, 2023; Lodge et al., 2023). These concerns are particularly relevant to learning procrastination, as the availability of GenAI may paradoxically reinforce avoidance behaviors when students use it to bypass rather than support their learning efforts.

On the positive side, large-scale surveys have documented high adoption rates among university students, with a notable increase over recent years, and identified factors that are associated with acceptance, including perceived usefulness, accessibility, and individual factors such as gender, digital literacy, and ethical awareness (Cabrera & Neville, 2025; Katsantonis & Katsantonis, 2024; Sergeeva et al., 2025; Shoufan, 2023; Ivanov et al., 2024; Strzelecki, 2023). The TPB has been widely applied to examine GenAI adoption, with studies indicating that attitudes, subjective norms, and perceived behavioral control are significantly associated with behavioral intentions (Ivanov et al., 2024; Zhao et al., 2024), and that integrated models combining TPB with unified theory of acceptance and use of technology or technology acceptance model are associated with variations in actual usage (Venkatesh et al., 2003). AI anxiety has received growing attention; research has developed validated anxiety scales and indicated that AI anxiety is negatively associated with learning motivation and is associated with changes in the perceived usefulness–intention relationship (Y. Y. Wang & Wang, 2022; Y. M. Wang et al., 2024). Concurrently, the broader learning procrastination literature has extensively documented that approximately half of university students self-identify as procrastinators, with robust links to poorer academic performance and psychological distress (Steel, 2007; Klingsieck et al., 2013; Alaya et al., 2021).

Despite these advances, several important gaps remain. Few studies have directly examined the association between students’ actual GenAI use for specific learning tasks and procrastination, as most work has focused on acceptance or intentions rather than behavioral outcomes. Moreover, although TPB has been validated for technology adoption, its application to procrastination in GenAI-assisted learning—particularly the indirect pathways through behavioral attitude, subjective norm, perceived behavioral control, and behavioral intention—remains underexplored. Furthermore, AI anxiety has not been systematically tested as a boundary condition in the intention–behavior gap, leaving the moderating role of learning GenAI anxiety (or similar constructs) unclear. The present study addresses these gaps by integrating TPB to examine (a) the direct association between GenAI use and GALP, (b) the indirect associations of behavioral attitude, subjective norm, perceived behavioral control, and behavioral intention with GALP through behavioral intention, which offers a contextual extension of TPB and procrastination research into the GenAI domain, and (c) the correlation of learning GenAI anxiety with the behavioral intention–GALP association. Table 1 summarizes these gaps and contributions.

### 2.2. GenAI-Assisted Learning Procrastination and the Theory of Planned Behavior

From the perspective of intention–behavior consistency, procrastination can be understood as the avoidance of goal-directed behavior, reflecting a discrepancy between intention and actual action (Steel, 2007). This view aligns with the TPB, which has been widely used to explain behavior-related outcomes by examining the relationships among behavioral beliefs, perceived social expectations, perceived control, behavioral intention, and behavior. Within the TPB, behavioral intention is regarded as the most proximal motivational factor associated with behavior, while behavioral attitude, subjective norm, and perceived behavioral control are theorized to shape behavioral intention (Ajzen, 1991). These belief-related constructs may also be indirectly associated with behavior-related outcomes through behavioral intention (Ajzen & Driver, 1992a; Armitage & Christian, 2003). In the context of GenAI-assisted learning, this logic suggests that students’ procrastination in using GenAI for learning tasks may be understood through a similar TPB-based pattern of associations. However, a conceptual caveat is in order: not all delayed or avoided use of GenAI should be equated with procrastination. Students might deliberately choose not to use GenAI due to pedagogical concerns, academic integrity considerations, task inappropriateness, or instructor guidance—decisions that represent reasoned, autonomous choices rather than maladaptive delay. With this distinction in mind, in this study, GALP is specifically defined as the unnecessary delay in initiating or completing the use of GenAI to support learning tasks (e.g., daily assignments, final-term assignments, and examination preparation), despite holding a genuine intention to use the technology and being aware of the potential costs of such delay. This specification distinguishes GALP from the general non-use or conscious avoidance of GenAI, as it centers on the intention–behavior gap in the context of GenAI-assisted learning. Applying the TPB logic to this specific form of procrastination, this study examines whether students’ evaluations, perceived peer expectations, and perceived control regarding GenAI use are associated with behavioral intention, and whether behavioral intention, in turn, is associated with GALP. Specifically, the more positively an individual views a behavior, the more they perceive social pressure to perform it, and the greater their sense of control over it, the stronger their intention tends to be, and stronger intention is in turn associated with a greater likelihood of actual performance. Within this process, behavioral intention serves as an indirect association between the aforementioned three variables and behavior (Armitage & Christian, 2003).

Behavioral attitude refers to an individual’s favorable or unfavorable evaluation of performing a specific behavior. When an individual holds a positive attitude towards a given task, the likelihood of performing it increases, and procrastination decreases (Xue et al., 2023). In procrastination research, prior studies have shown that more positive evaluations of a task, including perceived importance, priority, interest, and enjoyment, are generally associated with lower levels of procrastination (Claessens et al., 2010; Fang et al., 2024; Pychyl et al., 2000). In this study, behavioral attitude refers to students’ favorable or unfavorable evaluation of using GenAI to complete daily assignments, final-term assignments, and examination preparation. When students evaluate GenAI-assisted learning task completion more positively, they may be more willing to engage in this behavior and less likely to postpone it.

Subjective norm refers to students’ perceived social pressure to use GenAI to complete learning tasks. Classmates and peer norms may be associated with procrastination-related behavior. For example, when students clearly perceive a shared norm of completing tasks early, they tend to report lower levels of procrastination (Ackerman & Gross, 2005). In this study, subjective norm is operationalized as perceived peer expectations, given that classmates are a salient reference group in university learning contexts. This is especially relevant in the Chinese higher education context, where academic competition, peer comparison, and achievement-oriented expectations may make students attentive to classmates’ views and practices regarding GenAI-assisted learning.

Perceived behavioral control refers to an individual’s subjective assessment of the ease or difficulty of performing a specific behavior (Ajzen & Driver, 1992a; Bamberg et al., 2003). Existing studies have shown that procrastination decreases when students possess higher self-efficacy, a greater sense of control, greater autonomy, or a more internal locus of control in relation to learning tasks (B. Gao et al., 2025; Li et al., 2022; Santos et al., 2025; Tan et al., 2025; Tian et al., 2023; To et al., 2021; Gökalp & Haktanir, 2024). In this study, perceived behavioral control refers to students’ perceived capability and controllability in using GenAI to complete learning tasks. Students who feel more capable of and in control of completing GenAI-assisted learning tasks may be less likely to postpone such use.

Behavioral intention is a conceptually defined construct associated with action, reflecting an individual’s willingness to exert effort to achieve a goal. Behavioral intention is negatively associated with procrastination. Research on implementation intentions and goal pursuit has shown that clearer intentions are associated with higher rates of task completion and lower procrastination, partially because they facilitate the initiation of action, resistance to distractions, and recovery from setbacks (Gollwitzer & Sheeran, 2006). Results of an experimental study further indicated that when university students were prompted to form explicit behavioral intentions, they were substantially more likely to complete tasks as planned than the control group (Owens et al., 2008). In this study, behavioral intention refers to students’ readiness and willingness to use GenAI to complete learning tasks. Stronger behavioral intention is therefore associated with lower GALP.

Based on the above reasoning, the following research hypotheses are proposed:

**H1.**  *Behavioral intention in completing GenAI-assisted learning task is negatively associated with GALP.*

**H2.**  *Behavioral attitude in completing GenAI-assisted learning task is positively associated with behavioral intention.*

**H3.**  *Behavioral attitude in completing GenAI-assisted learning task is negatively associated with GALP.*

**H4.**  *Subjective norm in completing GenAI-assisted learning task is positively associated with behavioral intention.*

**H5.**  *Subjective norm in completing GenAI-assisted learning task is negatively associated with GALP.*

**H6.**  *Perceived behavioral control in completing GenAI-assisted learning task is positively associated with behavioral intention.*

**H7.**  *Perceived behavioral control in completing GenAI-assisted learning task is negatively associated with GALP.*

### 2.3. GenAI Use, Theory of Planned Behavior Constructs, and GenAI-Assisted Learning Procrastination

In recent years, GenAI has played a significant role in higher education. University students use GenAI for a range of learning-related activities, including information retrieval, idea generation, code generation, text drafting, and academic writing support (Stojanov, 2023; Strzelecki, 2023). However, the association between GenAI use and procrastination is theoretically ambivalent. On the one hand, GenAI use is theoretically associated with lower perceived difficulty in learning tasks, as it provides immediate feedback, idea generation, language support, and procedural guidance—features that may facilitate task initiation and completion. On the other hand, GenAI use has also been associated with overreliance, superficial engagement, academic misconduct, or avoidance of cognitively demanding work. Therefore, GenAI use is not necessarily associated with reduced procrastination (Abbas et al., 2024; Cui et al., 2025). Its association with GALP may depend on the psychological beliefs and intentions that guide students’ integration of GenAI into their learning activities.

Based on the task-facilitation pathway, this study hypothesizes that students’ use of GenAI for learning task completion is associated with more favorable attitudes, stronger perceived peer norms, greater perceived behavioral control, stronger behavioral intention, and lower GALP. Prior studies have suggested that GenAI applications in higher education can support personalized learning pathways, adaptive feedback, and learning process optimization (Alqahtani et al., 2023; Z. Gao et al., 2024; Gupta et al., 2023). Such experiences may be associated with more positive evaluations of GenAI-assisted learning and greater recognition of GenAI as a useful learning resource among students (Liang & Mao, 2025; Molino et al., 2020).

GenAI use may also be associated with students’ perceived social norms. Research on GenAI adoption in higher education suggests that students’ use of GenAI may be shaped by peers’, instructors’, and other relevant social groups’ attitudes, expectations, and practices (Ivanov et al., 2024). In the present study, because subjective norm is operationalized as perceived peer expectation, students who use GenAI more frequently may also be more likely to perceive GenAI-assisted learning as a practice recognized or expected within their peer environment.

In addition, GenAI use may be associated with perceived behavioral control and behavioral intention. Previous research has demonstrated that GenAI is associated with resources that help students cope with learning challenges, which may be associated with reduced self-doubt and greater perceived behavioral control (Xie & Wang, 2025). An observational study from the perspective of teachers also indicated that GenAI use was associated with a stronger tendency to complete learning tasks on time, which in turn was associated with lower levels of procrastination (Hasanein & Sobaih, 2023). These findings collectively suggest that students’ use of GenAI for learning task completion may be positively associated with perceived control and behavioral intention, both of which may in turn be associated with lower GALP.

Based on the above reasoning, the following research hypotheses are proposed:

**H8.**  *Students’ use of GenAI for learning task completion is positively associated with behavioral attitude toward GenAI-assisted learning task completion.*

**H9.**  *Students’ use of GenAI for learning task completion is positively associated with subjective norm regarding GenAI-assisted learning task completion.*

**H10.**  *Students’ use of GenAI for learning task completion is positively associated with perceived behavioral control over GenAI-assisted learning task completion.*

**H11.**  *Students’ use of GenAI for learning task completion is positively associated with behavioral intention to use GenAI for learning task completion.*

**H12.**  *Students’ use of GenAI for learning task completion is negatively associated with GALP.*

### 2.4. Learning GenAI Anxiety as a Moderator of the Intention–Procrastination Association

With the rapid development of technology, increasing attention has been paid to AI anxiety. Early studies on technology-related anxiety can be traced back to the first generation of computers, and note that computer-related anxiety was partly rooted in concerns that computers might threaten human significance or reduce individuals’ sense of control (Marcoulides, 1989; Charlton & Birkett, 1995; Barbeite & Weiss, 2004). With the rapid advancement of GenAI, such anxiety has become increasingly salient in educational contexts (Darancik et al., 2025). AI anxiety has been associated with inaccurate perceptions of technological development, confusion about autonomy, and sociotechnical blindness and is defined as feelings of fear or agitation about out-of-control AI (Johnson & Verdicchio, 2017).

For university students, learning to use GenAI may involve unfamiliar functions, complex algorithms, and rapidly changing technological applications, which may be associated with anxiety for many learners (Y. M. Wang et al., 2024). Learning GenAI anxiety, as a specific component of AI anxiety, refers to anxiety related to learning AI technologies and has been shown to be relevant to technology-related learning behavior (Y. Y. Wang & Wang, 2022). Prior studies on technology anxiety have also suggested that anxiety may be associated with the relationship between intention and subsequent learning activities, particularly in terms of whether individuals act on their intentions when facing technological demands or uncertainty (Brosnan & Lee, 1998; Russon et al., 1994; Y. S. Wang, 2007).

The present study specifies learning GenAI anxiety as a moderator of the association between behavioral intention and GALP, rather than of the earlier belief-formation paths. This specification is grounded in the intention–behavior gap literature, which suggests that even strong behavioral intention may fail to translate into corresponding action when individuals encounter emotional, contextual, or self-regulatory barriers (Sheeran & Webb, 2016). In the context of GenAI-assisted learning, learning GenAI anxiety may be particularly relevant at this point, as anxiety may shape whether students act on their intentions or delay using GenAI for learning tasks. Thus, learning GenAI anxiety may function as a boundary condition in the association between behavioral intention and GALP.

Importantly, the moderating role of learning GenAI anxiety should not be understood only as a simple weakening effect. On the one hand, anxiety may be negatively associated with the translation of intention into timely GenAI-assisted learning actions, which may be associated with increased hesitation, avoidance, or perceived difficulty. On the other hand, when students experience higher learning GenAI anxiety, behavioral intention may be more strongly associated with action, because stronger intention may be associated with overcoming avoidance tendencies and engaging in GenAI-assisted learning despite anxiety. Therefore, this study examines whether learning anxiety about GenAI moderates the association between behavioral intention and GALP. Based on the above reasoning, the following research hypothesis is proposed:

**H13.**  *Learning GenAI anxiety moderates the association between behavioral intention to use GenAI for learning task completion and GALP.*

**Figure 1:**
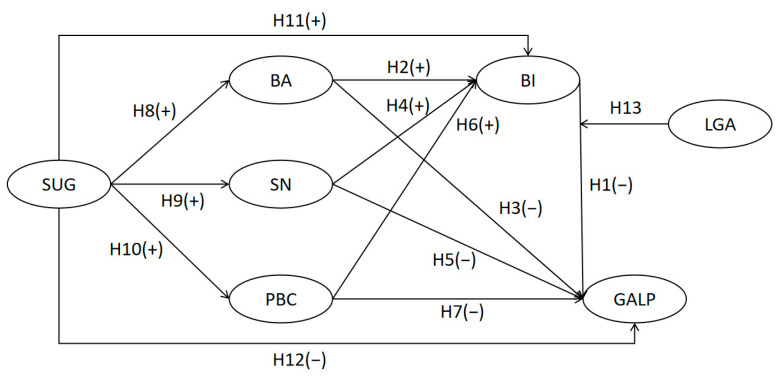
Hypothesized model.

The hypothesized model of this study is shown in Figure 1.

## 3. Method

### 3.1. Participants

Participants were undergraduate students recruited from three universities in Beijing, China. All three institutions are “Project 985” universities, representing the higher tier of Chinese higher education. The use of elite university students offers a distinct advantage: it minimizes the confounding effects of basic digital literacy and language comprehension on responses, allowing for a cleaner examination of the psychological mechanisms underlying GALP. Participants were recruited through voluntary response sampling. Recruitment announcements were posted on the online communication platforms of the three universities, and students who were willing to participate clicked the link to complete the questionnaire. Data were collected through a cross-sectional questionnaire survey between 5 March and 9 March 2026. Before completing the questionnaire, all participants were informed of the research purpose, assured that their participation was voluntary and anonymous, and provided written informed consent.

A total of 1395 questionnaires were collected. To ensure data quality, responses were excluded if participants selected the same response option across all items. After this screening procedure, 1243 valid questionnaires remained, yielding an effective response rate of 89.10%.

Among the valid respondents, 601 were male, and 642 were female. In terms of field of study, 358 students were from humanities and social sciences, 454 from science and engineering, 276 from medical sciences, and 155 from agricultural sciences. Regarding grade level, 294 were first-year students, 295 were second-year students, 356 were third-year students, and 298 were fourth-year students.

### 3.2. Measurements

The variables measured in this study included students’ use of GenAI, behavioral attitude, subjective norm, perceived behavioral control, behavioral intention, GALP, and learning GenAI anxiety. Except for learning GenAI anxiety, all variables were measured using a five-point Likert scale ranging from 1 = “strongly disagree” to 5 = “strongly agree.” Learning GenAI anxiety was measured using a seven-point Likert scale ranging from 1 = “strongly disagree” to 7 = “strongly agree.” All self-developed scales were constructed with reference to the same focal behavior, namely GenAI-assisted learning task completion, defined as students’ use of GenAI to support daily assignments, final-term assignments, and examination preparation.

The students using GenAI, behavioral attitude, subjective norm, perceived behavioral control, behavioral intention, and GALP items were developed for the present study based on the focal behavior specified above.

To establish the content validity of the newly developed scales, the initial item pool was generated based on theoretical definitions of each construct (Ajzen, 1991). The draft items were then reviewed by a panel of three experts in educational psychology and survey methodology, who were asked to evaluate each item for relevance, clarity, and representativeness to the target construct. Based on their feedback, we removed several items (e.g., “I think using GenAI to complete daily assignments is urgent”) to ensure they adequately covered the conceptual domain of each construct.

To examine item clarity, comprehensibility, and appropriateness from the perspective of the target respondent population, a pilot test was conducted prior to the formal survey. A sample of 20 undergraduate students (10 male, 10 female) from a university in Beijing, who were not included in the final sample, participated in the pilot test. Participants completed the draft questionnaire and were then invited to provide feedback on item wording, clarity, and relevance through a brief debriefing session. No additional items were deleted, as all remaining items demonstrated acceptable clarity and relevance based on pilot participant feedback.

To report the questionnaire in English, a translation and back-translation procedure was conducted. The original Chinese items were translated into English by a bilingual researcher and then independently back-translated into Chinese by another bilingual researcher who was not familiar with the original wording. Discrepancies were discussed and resolved through consensus.

Students’ use of GenAI for learning task completion (SUG) was measured with three items assessing how frequently students used GenAI to support daily assignments, final-term assignments, and examination preparation. Behavioral attitude (BA) was measured with nine items assessing students’ evaluations of using GenAI for learning task completion, including perceived importance, value, and interest. Subjective norm (SN) was measured with three items assessing students’ perceived peer expectations regarding the use of GenAI for learning task completion. Perceived behavioral control (PBC) was measured with nine items assessing students’ perceived capability and controllability in using GenAI to complete learning tasks. The items covered confidence in using GenAI, perceived control over the use process, and perceived autonomy in deciding whether to use GenAI. Behavioral intention (BI) was measured with six items assessing students’ intention and willingness to use GenAI for learning task completion. GenAI-assisted learning procrastination (GALP) was measured using three items that assessed students’ tendency to delay or postpone using GenAI for learning tasks, despite a genuine intention to do so. Consistent with the conceptual definition above, the scale items were designed to capture unnecessary delay in the presence of intention, rather than mere non-use or deliberate avoidance. The standardized factor loadings, Cronbach’s α, composite reliability (CR), and average variance extracted (AVE) for all constructs are reported in Table 2.

To examine whether the scales functioned equivalently across disciplinary contexts, we conducted multigroup confirmatory factor analysis (MG-CFA) across four groups (humanities and social sciences, n = 358; science and engineering, n = 454; medical sciences, n = 276; agricultural sciences, n = 155). We tested configural, metric, and scalar invariance for each scale. For GALP (3 items), full scalar invariance was supported (CFI = 1.000, RMSEA = 0.000 at configural; ΔCFI = 0.002 at scalar). For SUG (3 items), full scalar invariance was also supported (CFI = 1.000, RMSEA = 0.000; ΔCFI = 0.000). For the TPB constructs (BA, SN, PBC, BI; 27 items), the configural model showed acceptable fit (CFI = 0.892, RMSEA = 0.064). Both metric (ΔCFI = 0.001) and scalar (ΔCFI = −0.001) models met the invariance criteria, supporting full scalar invariance across the four groups. These results indicate that all scales measured the same constructs across students from different disciplinary backgrounds.

Learning GenAI anxiety (LGA) was measured with eight items adapted from the AI anxiety scale developed by Y. Y. Wang and Wang (2022). The items were adapted to the GenAI learning context and assessed students’ anxiety about learning to understand, use, interact with, and keep up with GenAI techniques or products. The adapted Chinese version was used in the formal survey, and the English wording reported in this manuscript was obtained through the translation and back-translation procedure described above. The standardized factor loadings, Cronbach’s α, CR, and AVE for LGA are also reported in Table 2. The complete list of items for all scales is provided in Appendix A.

The standardized factor loadings ranged from 0.58 to 0.95. Cronbach’s α values ranged from 0.83 to 0.95, indicating acceptable to excellent internal consistency. Composite reliability (CR) values ranged from 0.83 to 0.95, exceeding the recommended threshold of 0.70. Average variance extracted (AVE) values ranged from 0.44 to 0.87. Although the AVE values for BA, PBC, and BI were slightly below 0.50, their CR values were all above 0.80, suggesting acceptable convergent validity according to Fornell’s criterion (Fornell & Larcker, 1981).

A confirmatory factor analysis was conducted to examine the seven-factor measurement model, in which SUG, BA, SN, PBC, BI, GALP, and LGA were specified as distinct latent constructs. The model showed acceptable fit: χ^2^/df = 3.55, CFI = 0.928, TLI = 0.922, RMSEA = 0.045, and SRMR = 0.032. These results provide support for the construct validity of the measurement model.

### 3.3. Data Analysis

SPSS version 27.0 was used to conduct Cronbach’s alpha reliability tests, common method bias tests, descriptive statistical analyses, and correlation analyses of the questionnaire. Mplus version 8.3 was used to estimate standardized factor loadings, perform confirmatory factor analysis for the measurement model, and conduct path analysis for the structural model. A multigroup confirmatory factor analysis was also conducted in Mplus to test measurement invariance across the four disciplinary groups. Based on the Mplus output, Excel was used to calculate the composite reliability (CR) and average variance extracted (AVE) for each variable. Since the model contains interaction terms, the MLR estimator and ALGORITHM = INTEGRATION were employed for parameter estimation. The latent variable interaction method was adopted, and the interaction term BI × LGA was constructed using the XWITH command, with Mplus automatically centering the latent variables. Gender, grade, and major were included as control variables in the model.

## 4. Results

### 4.1. Common Method Bias Tests

Because all variables were measured using self-report questionnaires from the same participants, common method bias was examined using Harman’s single-factor test. The first factor explained 29.39% of the total variance, which was below the commonly used threshold of 40%. Therefore, Harman’s single-factor test did not indicate a serious single-factor problem. However, given the limitations of this test, the results should still be interpreted with caution.

### 4.2. Descriptive Statistical Analysis

Descriptive statistics for all variables are presented in Table 3. As shown in Table 3, the mean score of GALP was 2.60 on a five-point scale, and the mean score of LGA was 3.40 on a seven-point scale. The mean scores of SUG, BA, SN, PBC, and BI were slightly above the midpoint of the five-point scale.

### 4.3. Correlation Analysis

As shown in Table 4, SUG, BA, SN, PBC, and BI were positively correlated with one another. GALP was negatively correlated with SUG, BA, SN, PBC, and BI. LGA showed small but significant correlations with the other variables.

### 4.4. Path Analysis

Gender, grade, and major were included as control variables. A structural equation model was employed to examine the path relationships among the variables. As shown in Table 5, only the science and engineering major had a significant negative association with PBC (β = −0.114, *p* = 0.020).

Before testing the moderated mediation model, this study first examined the direct paths among variables. As shown in Table 4, all direct paths were statistically significant. Specifically, SUG was positively associated with BA, SN, PBC, and BI, which was consistent with H8–H11. BA, SN, PBC, and BI were negatively associated with GALP, which was consistent with H1, H3, H5, and H7. SUG was also negatively associated with GALP, which was consistent with H12. The direct association between SUG and GALP remained significant, consistent with indirect associations.

Before examining the path coefficients, we evaluated the structural model’s fit. The model showed acceptable fit: χ^2^/df = 2.95, CFI = 0.929, TLI = 0.922, RMSEA = 0.040, and SRMR = 0.032. These fit indices indicate that the hypothesized structural model adequately represented the observed data.

### 4.5. Moderated Indirect Association Analysis

Before testing the moderated indirect association model, this study also examined the indirect associations between SUG and GALP. The results are presented in Table 6. All indirect associations were significant. Specifically:

Indirect path from SUG to GALP through BA and BI: SUG → BA → BI → GALP was significant.

Indirect path from SUG to GALP through SN and BI: SUG → SN → BI → GALP was significant.

Indirect path from SUG to GALP through PBC and BI: SUG → PBC → BI → GALP was significant.

Indirect path from SUG to GALP through BI only: SUG → BI → GALP was significant.

The total indirect association between SUG and GALP was significant, as was the total association between SUG and GALP. These results are statistically consistent with the proposed Theory of Planned Behavior-based indirect association model.

The model explained 58.5% of the variance in BI (R^2^ = 0.585) and 71.6% of the variance in GALP (R^2^ = 0.716). These values indicate that the TPB constructs collectively explain a substantial proportion of the variance in students’ behavioral intention and procrastination in GenAI-assisted learning, with the model accounting for nearly three-quarters of the variance in GALP.

The standardized total association of SUG on GALP was −0.278 (*p* < 0.001). The indirect association accounted for approximately 30.9% of this total association (standardized indirect association = −0.086, *p* < 0.01), suggesting that the indirect associations through TPB constructs accounted for a meaningful proportion of the total association.

Because the model included a latent interaction term (BI × LGA), the MLR estimator and the ALGORITHM = INTEGRATION command were used in Mplus for parameter estimation. The latent interaction term BI × LGA had a significant negative association with GALP (*b* = −0.137, 95%CI: [−0.172, −0.102], *p* < 0.001), indicating that the BI-GALP association varied by LGA level. This result was consistent with H13.

**Figure 2:**
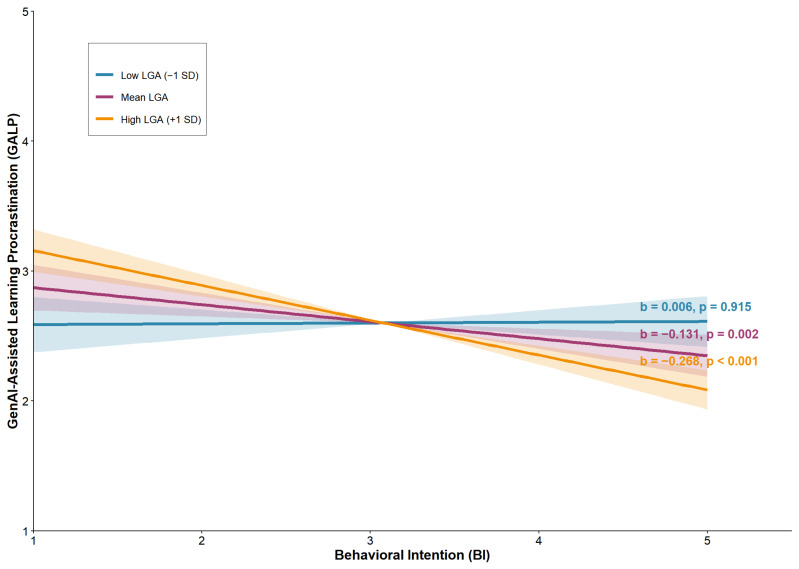
Simple slopes of the association between BI and GALP at different levels of LGA.

A simple slope analysis was conducted to further investigate the moderating effect. The moderating variable LGA was divided into high, middle, and low groups, defined as one standard deviation above and below the mean, to examine the association between BI and GALP across LGA levels. The simple slope plot is shown in Figure 2.

Simple slope analyses showed that the association between BI and GALP was not significant at low LGA (*b* = 0.006, *SE* = 0.052, *t* = 0.106, *p* = 0.915), but was significant and negative at mean LGA (*b* = −0.131, *SE* = 0.043, *t* = −3.037, *p* = 0.002) and high LGA (*b* = −0.268, *SE* = 0.040, *t* = −6.630, *p* < 0.001). These results indicate that the negative association between BI and GALP was stronger among students with higher LGA levels.

The product-of-coefficients method was used to test the moderated indirect association indices, and the results are presented in Table 7. All four specific indices were negative and statistically significant, indicating that the indirect associations of SUG on GALP through BA, SN, PBC, and BI were stronger when LGA was high. The total moderated indirect association index was also significant (estimate = −0.088, *p* < 0.001).

## 5. Discussion

### 5.1. University Students’ Use of GenAI and GenAI-Assisted Learning Procrastination

The present study found that students’ use of GenAI was negatively associated with GALP, suggesting that more frequent users tend to experience a narrower intention–behavior gap in GenAI-assisted learning. A plausible explanation is that accumulated experience with GenAI reduces the psychological friction of task initiation. Students who use GenAI more often are likely to have developed greater familiarity with its capabilities and limitations, established routines for integrating it into their workflow, and lower uncertainty about how to begin. In contrast, less experienced students may hesitate about what to expect or whether the tool will be helpful—uncertainty that can lead to unnecessary delays. Prior research has shown that students commonly use GenAI for idea generation, drafting, and clarifying their thinking (Alqasham, 2023; Barrett & Pack, 2023; Boubker, 2024; Chan & Hu, 2023; Dahri et al., 2025; Dai et al., 2023). Over time, such repeated engagement may lower the cognitive cost of initiating GenAI-assisted work, making it easier to promptly follow through on intentions.

This negative association should not be interpreted as evidence that GenAI use is uniformly beneficial for learning. Lower GALP does not necessarily reflect deeper cognitive engagement, higher learning quality, or responsible use—indeed, using GenAI as a substitute for active thinking may foster overreliance or superficial processing. More importantly, it should not be equated with pedagogically appropriate use. In many learning scenarios, students may deliberately limit or avoid GenAI to cultivate independent problem-solving skills, comply with academic integrity policies, or because the task calls for creative or reflective work that resists algorithmic assistance. Such intentional non-use is qualitatively distinct from procrastination (i.e., delay despite intention), and it was not captured by our GALP measure. Thus, the negative association between SUG and GALP should be understood as a specific behavioral pattern rather than a general endorsement of GenAI use in higher education.

### 5.2. Indirect Associations Through Behavioral Attitude, Subjective Norm, Perceived Behavioral Control, and Behavioral Intention

The results were statistically consistent with indirect associations linking students’ use of GenAI and GALP through behavioral attitude, subjective norm, perceived behavioral control, and behavioral intention.

Students’ use of GenAI was associated with more favorable attitudes toward GenAI-assisted learning, which in turn were associated with lower GALP. One plausible explanation is that students who hold positive evaluations of GenAI are less likely to experience ambivalence or hesitation when the moment comes to act on their intention to use it. Prior research has shown that students generally recognize the auxiliary value of GenAI tools, holding favorable views of their usefulness despite being aware of their limitations (Shoufan, 2023). Such favorable attitudes may reduce the psychological friction that often delays task initiation, making it easier for students to follow through on their intentions promptly (Tossell et al., 2024).

Students’ use of GenAI was also associated with stronger perceived peer expectations for GenAI-assisted learning, which in turn were associated with lower GALP. In this study, subjective norm was operationalized as perceived peer expectations rather than broader social norms. Students who use GenAI more frequently tend to perceive stronger peer-based expectations for completing GenAI-assisted learning tasks. Such peer norms may serve as a social reference point that clarifies what constitutes appropriate and timely behavior, thereby reducing uncertainty about whether and when to act on one’s intention to use GenAI. This interpretation aligns with prior research emphasizing the role of social influence in shaping technology-related behavior (Venkatesh et al., 2003).

Students’ use of GenAI was associated with greater perceived behavioral control over GenAI-assisted learning, which in turn was associated with lower GALP. Students who feel more capable of and in control of using GenAI for learning tasks may encounter less hesitation when translating their intention into action, as they are more confident about how to proceed and less uncertain about potential difficulties. This finding is consistent with prior research suggesting that GenAI can support students’ self-regulated learning and perceived control in technology-enhanced learning environments (Ng et al., 2024). From the perspective of self-regulated learning (Zimmerman, 2002), GenAI may enhance perceived behavioral control by helping students clarify learning goals, obtain step-by-step guidance when encountering difficulties, and use feedback to adjust their strategies (Ajlouni et al., 2023; Alqasham, 2023; Chan & Lee, 2023; Ng et al., 2024).

The results also showed that behavioral intention was involved in the indirect association between students’ use of GenAI and GALP. Students who used GenAI more frequently tended to report greater readiness and willingness to use GenAI for learning and task completion and were less likely to postpone such use. This finding suggests that more frequent use may strengthen the motivational commitment to act on one’s intentions, thereby narrowing the intention–behavior gap that characterizes GALP. This interpretation is consistent with Chiu’s (2024) argument that GenAI is associated with personalized learning pathways and collaborative environments that support students’ needs for autonomy, competence, and relatedness, which, in turn, correspond to a stronger readiness to engage in learning tasks with GenAI rather than to postpone them (Chiu, 2024).

### 5.3. Theory of Planned Behavior-Based Indirect Associations Through Behavioral Intention

Beyond the separate indirect associations of behavioral attitude, subjective norm, perceived behavioral control, and behavioral intention, the results were also statistically consistent with the TPB-based proposition that these belief-related constructs are associated with behavior-related outcomes through behavioral intention (Ajzen, 1991; Ajzen & Driver, 1992b; Armitage & Christian, 2003). In this study, students’ use of GenAI for learning task completion was associated with more favorable evaluations of GenAI-assisted learning, stronger perceived peer expectations, and greater perceived control over GenAI use. These belief-related constructs, in turn, were associated with stronger behavioral intentions, which were associated with lower levels of GALP. In other words, students who use GenAI more frequently tend to hold more favorable attitudes, perceive stronger peer norms, and feel greater control over GenAI use; these beliefs are associated with stronger intentions, which in turn are associated with a narrower intention–behavior gap—the hallmark of GALP.

### 5.4. The Moderating Effect of Learning GenAI Anxiety

The results showed that learning GenAI anxiety moderated the association between behavioral intention and GALP. Specifically, the negative association between behavioral intention and GALP was less pronounced among students with low learning GenAI anxiety levels but stronger among those with high learning GenAI anxiety levels.

This finding differs somewhat from perspectives suggesting that state anxiety may be associated with procrastination as an emotion regulation strategy to alleviate psychological discomfort (Spielberger, 1966; Tang et al., 2015). Here, however, under high anxiety, the negative association between behavioral intention and GALP was stronger rather than weaker. One plausible interpretation is that anxiety, in this context, functions not as a simple barrier to action but as a motivational amplifier, making behavioral intentions more consequential. When students experience high anxiety about using GenAI, the decision to act on their intentions requires greater cognitive and emotional effort. Under such conditions, a strong intention becomes a critical psychological resource, helping students overcome avoidance tendencies and initiate action despite discomfort. This interpretation aligns with research suggesting that anxiety can enhance goal-directed behavior when individuals possess clear implementation intentions (Gollwitzer & Sheeran, 2006) or when the behavior is perceived as instrumentally valuable.

This pattern can be further understood through the lens of self-regulation theory. Anxiety signals the presence of a psychologically significant threat or challenge, which increases the perceived costs of inaction (Carver & Scheier, 1998). When students experience high anxiety about using GenAI, the decision to delay or avoid GenAI-assisted learning is itself anxiety-provoking because procrastination does not eliminate the source of anxiety but merely postpones confronting it. Under such conditions, a strong behavioral intention may serve as a commitment device, helping students tolerate the discomfort associated with task engagement and enabling them to act despite anxiety rather than postponing action to escape it (Sirois & Pychyl, 2013). In other words, high anxiety raises the psychological stakes of both action and inaction; when intentions are strong enough to tip the balance toward action, behavior becomes more likely than when anxiety is low, and intentions are less necessary. This interpretation helps explain why the behavioral intention–GALP association was stronger among high-anxiety students in the present sample.

Taken together, these findings suggest that learning GenAI anxiety serves as a “moderating catalyst” that amplifies the behavioral relevance of intentions, rather than as a simple barrier to technology use.

## 6. Conclusions and Implications

### 6.1. Conclusions

This study examines the association between students’ use of GenAI for learning task completion and GALP within the TPB framework among 1243 Chinese university students. The findings show that students’ use of GenAI to complete learning tasks is negatively associated with GALP. The results are statistically consistent with TPB-based indirect associations linking students’ use of GenAI for learning task completion and GALP through behavioral attitude, subjective norm, perceived behavioral control, and behavioral intention. Specifically, students’ GenAI use is associated with more favorable attitudes toward GenAI-assisted learning, stronger perceived peer expectations, greater perceived behavioral control, and stronger behavioral intention, which, in turn, is associated with lower GALP. In addition, learning GenAI anxiety moderates the association between behavioral intention and GALP. The negative association between behavioral intention and GALP is stronger among students with higher learning GenAI anxiety levels. Given the cross-sectional design and the sample’s specific characteristics, these findings should be interpreted as conditional associations rather than causal evidence. As a contextual extension of TPB rather than a fundamental theoretical breakthrough, the contributions are best understood as initial empirical evidence, and their generalizability requires further investigation.

### 6.2. Theoretical Implications

Theoretically, this study contributes to the literature by specifying procrastination in the context of GenAI-assisted learning task completion. Rather than treating procrastination in learning as a general academic behavior, the study examined students’ tendency to delay or postpone the use of GenAI to complete specific learning tasks. This specification helps align the focal behavior, TPB constructs, and GALP within the same behavioral domain. More importantly, by defining GALP as unnecessary delay despite a genuine intention to use GenAI—rather than as mere non-use—this study clarifies a conceptual distinction that has been overlooked in prior research: not all non-use reflects procrastination, as students may deliberately choose to forgo GenAI for pedagogical or ethical reasons.

Second, the study offers a contextual extension of TPB’s application to GenAI-assisted learning rather than a fundamental reconceptualization of the theory. The findings suggest that the association between students’ use of GenAI for learning task completion and GALP can be understood through students’ behavioral attitude, subjective norm, perceived behavioral control, and behavioral intention regarding GenAI-assisted learning task completion. In essence, these indirect associations indicate that students’ beliefs and intentions regarding GenAI use are associated with the extent to which they successfully translate their intention to use GenAI into timely action—that is, the narrowing or widening of the intention–behavior gap that characterizes GALP. This provides a psychological perspective for understanding how students’ GenAI use is associated with procrastination-related outcomes in GenAI-assisted learning.

Third, the study highlights learning GenAI anxiety as an emotional boundary condition in the association between behavioral intention and GALP. Rather than treating anxiety only as a general barrier to technology use, the findings suggest that anxiety may be associated with the strength of the association between students’ intention to use GenAI and their procrastination in GenAI-assisted learning. Specifically, under high anxiety, the negative association between intention and procrastination was stronger, suggesting that anxiety may amplify rather than weaken the behavioral relevance of intentions. This counterintuitive pattern points to the value of integrating cognitive, motivational, and emotional perspectives in future research on GenAI-supported learning, particularly in understanding when and for whom intentions are more or less likely to translate into action.

### 6.3. Educational Practice Implications

The findings suggest that students’ use of GenAI for learning task completion is associated with lower levels of GALP, as it is linked to behavioral attitude, subjective norm, perceived behavioral control, and behavioral intention. In addition, learning GenAI anxiety moderated the association between behavioral intention and GALP, with the association being stronger among students with higher anxiety levels.

Before turning to practical implications, a conceptual caveat is in order. Lower GALP—that is, more timely use of GenAI—should not be equated with responsible or effective GenAI use. Timely use may simply reflect habitual or uncritical reliance on AI-generated outputs, which does not necessarily translate into meaningful learning. A growing body of research has therefore emphasized the importance of cultivating critical thinking in AI use—defined as a disposition to verify the source and content of AI-generated information, understand how AI models work and where they fail, and reflect on the broader implications of relying on AI (Lau et al., 2026). From this perspective, the educational goal is not merely to reduce procrastination, but to create conditions under which students can engage critically and reflectively with GenAI. Accordingly, the following implications center on fostering critical and reflective use of GenAI, with each recommendation grounded in the specific pathways identified in this study.

Fostering critical thinking through TPB-based pathways. The indirect associations identified in this study suggest that students’ GenAI use is associated with lower procrastination, partly through behavioral attitude, perceived behavioral control, and subjective norm, which, in turn, are associated with behavioral intention. To leverage these pathways, teachers could design activities that engage students in critically evaluating AI-generated content—for example, by comparing AI outputs with their own work, identifying inaccuracies or gaps, and articulating reasons for accepting, modifying, or rejecting AI-generated content. Such practices may help students develop more balanced attitudes toward GenAI, recognizing both its utility and its limitations. To strengthen perceived behavioral control, teachers could provide structured opportunities for students to practice critical evaluation skills in low-stakes settings before applying them to graded assignments. To shape peer norms, teachers could organize peer discussion groups where students share and critique their GenAI experiences, focusing on strategies for verifying and cross-checking AI-generated information.

Differentiated support based on learning GenAI anxiety. The moderating role of learning GenAI anxiety further suggests that differentiated support may be warranted. For students with higher anxiety, low-threshold skill training and guided practice may help reduce the psychological cost of GenAI-assisted learning, thereby facilitating the translation of intention into timely action. Critically, such support should embed critical thinking from the outset—teaching students not only how to use GenAI, but also how to question, verify, and contextualize its outputs. For students with lower anxiety, timely use may already come readily, yet without critical oversight, it may not translate into meaningful learning. Support for this group should therefore emphasize deeper critical engagement, responsible use, and reflection on when GenAI is and is not appropriate for specific learning tasks.

In summary, these implications are derived from cross-sectional associations and should be considered tentative, theory-informed directions rather than prescriptive interventions. Across all recommendations, critical thinking in AI use serves as the unifying thread: reducing GALP is valuable not as an end in itself, but as a means of creating conditions under which students can engage critically, reflectively, and responsibly with GenAI in their learning.

### 6.4. Research Limitations

Several limitations of this study should be acknowledged.

First, the research relied on cross-sectional survey data, which can show associations among variables but cannot determine directional relationships over time between students’ use of GenAI for learning task completion and changes in GALP. For example, students with lower initial tendencies toward procrastination may be more likely to adopt GenAI actively, suggesting the possibility of reverse causality. Future research employing longitudinal or experimental designs is needed to examine the temporal ordering of these associations.

Second, the generalizability of the findings is substantially constrained by the sampling strategy. All participants were recruited from three “Project 985” universities in Beijing—elite institutions in a highly developed metropolitan area with advanced technological infrastructure. This specific context has several important implications for generalizability. First, students at these elite universities are likely to have superior digital literacy and more equitable access to GenAI tools compared to students at non-elite institutions, which may amplify the observed associations. Second, Beijing, as the capital and a technological hub, provides an environment with widespread GenAI adoption and discourse, which may not be representative of other regions in China. Third, the competitive and achievement-oriented culture at elite universities may strengthen peer norms and behavioral intentions in ways that differ from other institutional contexts. Therefore, these findings should not be generalized to students at non-elite universities, vocational colleges, or institutions in less developed regions of China, where access to GenAI tools, digital literacy, and attitudes toward technology may differ substantially. Future research should include a broader range of institutions across different regions and tiers of Chinese higher education, as well as cross-cultural samples, to enhance generalizability.

Third, the use of voluntary response sampling introduces the possibility of self-selection bias. Students who chose to participate may have been more interested in or more familiar with GenAI than those who did not, which could have influenced the observed associations. Although no course credit or compensation was offered to minimize undue influence, self-selection remains a limitation of the recruitment method. Future research employing probability-based sampling strategies would help address this concern.

Fourth, the study did not fully examine the heterogeneous patterns across different forms of technology use, nor did it differentiate in detail between distinct learning contexts for GenAI application, such as routine assignments versus creative or reflective tasks, where different psychological mechanisms may underlie students’ GALP. Future research should explore these dimensions to better specify when and for whom the observed associations hold.

Fifth, reduced GALP should not be equated with improved learning outcomes. In the context of AI-assisted work, timely completion may reflect efficient support, but it may also reflect outsourcing, overreliance, or superficial engagement. Future research should include direct indicators of learning quality, cognitive engagement, academic integrity, or performance outcomes to provide a more comprehensive understanding of the educational implications of GenAI use.

Sixth, although the seven-factor measurement model showed acceptable fit and the reliability indices were generally adequate, several constructs were newly developed or adapted for the present study. Future research should further validate these scales across independent samples, different institutional contexts, and diverse student populations.

Seventh, although Harman’s single-factor test did not indicate a serious single-factor problem, this procedure cannot fully rule out common method variance. Future research should use procedural remedies, marker variables, multi-source data, multi-wave designs, longitudinal, experimental, or experience-sampling designs to better address this concern.

## Figures and Tables

**Table 1:** Summary of research gaps and the contribution of the prior studies.

Research Domain	What Prior Studies Have Done	What Remains Unresolved	Contribution of the Prior Studies
GenAI Acceptance & Use	Examined students’ perceptions, attitudes, and acceptance of GenAI; documented usage rates exceeding 77% among university students; identified gender, usage frequency, and ethical awareness as factors associated with attitudes (Cabrera & Neville, 2025; Katsantonis & Katsantonis, 2024; Sergeeva et al., 2025; Shoufan, 2023; Strzelecki, 2023).	Few studies have linked actual GenAI use with behavioral outcomes such as procrastination.	Examines the association between Students using GenAI and GALP.
TPB in Technology Contexts	Applied TPB to technology adoption and acceptance; validated TPB-based models for GenAI adoption (Ivanov et al., 2024; Zhao et al., 2024; Venkatesh et al., 2003).	Limited application to procrastination in GenAI-assisted learning.	Tests TPB-based indirect associations (behavioral attitude/subjective norm/perceived behavioral control → behavioral intention → GALP).
AI Anxiety & Learning Behavior	Developed AI anxiety scales and examined their association with learning behavior and motivation; indicated that AI anxiety is negatively associated with learning motivations (Y. Y. Wang & Wang, 2022; Y. M. Wang et al., 2024).	Learning GenAI anxiety as a boundary condition in the intention–behavior gap has not been examined.	Examines learning GenAI anxiety as a moderator of the association between behavioral intention and GALP.
Learning Procrastination	Extensively documented prevalence (approximately 50% of university students), antecedents (e.g., perfectionistic concerns), and consequences (e.g., poorer academic performance, anxiety, depression) (Steel, 2007; Klingsieck et al., 2013; Alaya et al., 2021).	Procrastination in GenAI-assisted learning contexts remains unexplored.	Defines and measures GALP as a context-specific form of procrastination.

**Table 2:** Standardized factor loadings, Cronbach’s α, composite reliability (CR), and average variance extracted (AVE) for all variables.

Variables	Items	Standardized Factor Loadings	Cronbach’s α	CR	AVE
SUG	SUG1	0.85	0.86	0.86	0.67
	SUG2	0.80			
	SUG3	0.81			
BA	BA1	0.67	0.88	0.88	0.44
	BA2	0.61			
	BA3	0.58			
	BA4	0.63			
	BA5	0.65			
	BA6	0.63			
	BA7	0.72			
	BA8	0.74			
	BA9	0.74			
SN	SN1	0.91	0.94	0.95	0.85
	SN2	0.92			
	SN3	0.93			
PBC	PBC1	0.68	0.89	0.88	0.46
	PBC2	0.66			
	PBC3	0.64			
	PBC4	0.65			
	PBC5	0.67			
	PBC6	0.67			
	PBC7	0.70			
	PBC8	0.71			
	PBC9	0.73			
BI	BI1	0.70	0.83	0.83	0.45
	BI2	0.72			
	BI3	0.64			
	BI4	0.65			
	BI5	0.64			
	BI6	0.64			
GALP	GALP1	0.91	0.95	0.95	0.87
	GALP2	0.95			
	GALP3	0.92			
LGA	LGA1	0.71	0.91	0.91	0.57
	LGA2	0.78			
	LGA3	0.77			
	LGA4	0.78			
	LGA5	0.75			
	LGA6	0.74			
	LGA7	0.75			
	LGA8	0.76			

**Table 3:** Descriptive statistical analysis of variables.

Variables	Minimum	Maximum	Mean	Standard Deviation
SUG	1.00	5.00	3.05	0.98
BA	1.11	4.89	3.08	0.81
SN	1.00	5.00	3.35	0.98
PBC	1.11	5.00	3.16	0.88
BI	1.00	5.00	3.08	0.90
GALP	1.00	5.00	2.60	0.97
LGA	1.00	7.00	3.40	1.51

**Table 4:** Correlation analysis among variables.

Variables	SUG	BA	SN	PBC	BI	GALP	LGA
SUG							
BA	0.594 **						
SN	0.456 **	0.437 **					
PBC	0.533 **	0.545 **	0.449 **				
BI	0.574 **	0.568 **	0.450 **	0.544 **			
GALP	−0.657 **	−0.651 **	−0.558 **	−0.675 **	−0.651 **		
LGA	0.100 **	0.133 **	0.065 *	0.088 *	0.170 **	−0.103 **	

Note: ** *p* < 0.01 (two-tailed); * *p* < 0.05 (two-tailed).

**Table 5:** Results of path analysis.

Hypotheses	Paths	*b*	β	*SE*	*p*	95%CI
H1	BI → GALP	−0.131	−0.137	0.043	<0.001	[−0.216, −0.047]
H2	BA → BI	0.310	0.273	0.062	<0.001	[0.190, 0.431]
H3	BA → GALP	−0.179	−0.164	0.046	<0.001	[−0.269, −0.090]
H4	SN → BI	0.110	0.116	0.035	<0.01	[0.040, 0.179]
H5	SN → GALP	−0.155	−0.171	0.027	<0.001	[−0.208, −0.101]
H6	PBC → BI	0.240	0.219	0.050	<0.001	[0.143, 0.337]
H7	PBC → GALP	−0.347	−0.331	0.048	<0.001	[−0.441, −0.253]
H8	SUG → BA	0.573	0.692	0.034	<0.001	[0.505, 0.642]
H9	SUG → SN	0.508	0.508	0.037	<0.001	[0.436, 0.581]
H10	SUG → PBC	0.530	0.617	0.034	<0.001	[0.463, 0.598]
H11	SUG → BI	0.280	0.297	0.053	<0.001	[0.176, 0.383]
H12	SUG → GALP	−0.176	−0.195	0.039	<0.001	[−0.252, −0.100]

**Table 6:** Results of indirect association analysis.

Indirect Association Paths	Estimate	*SE*	*p*	95%CI
Total Indirect Association (SUG → GALP)	−0.086	0.028	<0.01	[−0.139, −0.029]
SUG → BA → BI → GALP	−0.023	0.008	<0.01	[−0.040, −0.007]
SUG → SN → BI → GALP	−0.007	0.004	<0.05	[−0.014, −0.000]
SUG → PBC → BI → GALP	−0.017	0.007	<0.05	[−0.031, −0.003]
SUG → BI → GALP	−0.037	0.014	<0.01	[−0.064, −0.010]
Total Association (SUG → GALP)	−0.258	0.044	<0.001	[−0.346, −0.175]

**Table 7:** Moderated indirect association indices for the BI–GALP path moderated by LGA.

Moderated Indirect Association Paths	Estimate	*SE*	*p*	95%CI
SUG → BA → BI → GALP	−0.024	0.006	<0.001	[−0.035, −0.013]
SUG → SN → BI → GALP	−0.008	0.003	<0.01	[−0.013, −0.002]
SUG → PBC → BI → GALP	−0.017	0.004	<0.001	[−0.025, −0.009]
SUG → BI → GALP	−0.038	0.009	<0.001	[−0.055, −0.021]
Total Index of Moderated Indirect Association	−0.088	0.012	<0.001	[−0.111, −0.064]

Note. Each index represents the difference in the conditional indirect association between SUG and GALP via the specified indirect pathway at high (+1 SD) versus low (−1 SD) levels of LGA. The model includes the main association of LGA with GALP. LGA moderates only the BI–GALP association. All confidence intervals were obtained using the Delta method.

## Data Availability

Due to privacy restrictions, the data can be obtained from the corresponding author upon request.

## Abbreviations

The following abbreviations are used in this manuscript:

TPB	Theory of Planned Behavior
GALP	GenAI-assisted learning procrastination
SUG	Students using GenAI
BA	Behavioral attitude
SN	Subjective norm
PBC	Perceived behavioral control
BI	Behavioral intention
LGA	Learning GenAI anxiety

## Appendix A

**Table A1:** Measurement items for all variables.

Variables	Item Code	Item Wording
SUG	SUG1	I often use GenAI when completing daily assignments.
	SUG2	I often use GenAI when completing final-term assignments.
	SUG3	I often use GenAI when preparing for examinations.
BA	BA1	I think using GenAI to complete daily assignments is important.
	BA2	I think using GenAI to complete final-term assignments is important.
	BA3	I think using GenAI to prepare for examinations is important.
	BA4	I think using GenAI to complete daily assignments is valuable.
	BA5	I think using GenAI to complete final-term assignments is valuable.
	BA6	I think using GenAI to prepare for examinations is valuable.
	BA7	I think using GenAI to complete daily assignments is interesting.
	BA8	I think using GenAI to complete final-term assignments is interesting.
	BA9	I think using GenAI to prepare for examinations is interesting.
SN	SN1	Most of my classmates think I should use GenAI to complete daily assignments.
	SN2	Most of my classmates think I should use GenAI to complete final-term assignments.
	SN3	Most of my classmates think I should use GenAI to prepare for examinations.
PBC	PBC1	I am confident that I can use GenAI to complete daily assignments.
	PBC2	I am confident that I can use GenAI to complete final-term assignments.
	PBC3	I am confident that I can use GenAI to prepare for examinations.
	PBC4	I feel in control when using GenAI to complete daily assignments.
	PBC5	I feel in control when using GenAI to complete final-term assignments.
	PBC6	I feel in control when using GenAI to prepare for examinations.
	PBC7	Using GenAI to complete daily assignments is entirely up to me.
	PBC8	Using GenAI to complete final-term assignments is entirely up to me.
	PBC9	Using GenAI to prepare for examinations is entirely up to me.
BI	BI1	I intend to use GenAI to complete my daily assignments.
	BI2	I intend to use GenAI to complete my final-term assignments.
	BI3	I intend to use GenAI to prepare for my examinations.
	BI4	I am willing to exert effort to use GenAI to complete my daily assignments.
	BI5	I am willing to exert effort to use GenAI to complete my final-term assignments.
	BI6	I am willing to exert effort to use GenAI to prepare for my examinations.
GALP	GALP1	Although I plan to use GenAI for my daily assignments, I often unnecessarily put it off, even though I know it may cost me later.
	GALP2	Although I plan to use GenAI for my final-term projects, I often unnecessarily delay doing so, even though I know it may cost me later.
	GALP3	Although I intend to use GenAI for exam preparation, I often unnecessarily postpone it, even though I know it may cost me later.
LGA	LGA1	Learning to understand all of the special functions associated with a GenAI technique/product makes me anxious.
	LGA2	Learning to use GenAI techniques/products makes me anxious.
	LGA3	Learning to use specific functions of a GenAI technique/product makes me anxious.
	LGA4	Learning how a GenAI technique/product works makes me anxious.
	LGA5	Learning to interact with a GenAI technique/product makes me anxious.
	LGA6	Taking a class about the development of GenAI techniques/products makes me anxious.
	LGA7	Reading a GenAI technique/product manual makes me anxious.
	LGA8	Being unable to keep up with the advances associated with GenAI techniques/products makes me anxious.
